# Molecular cloning and transcriptional activity of a new *Petunia* calreticulin gene involved in pistil transmitting tract maturation, progamic phase, and double fertilization

**DOI:** 10.1007/s00425-013-1971-4

**Published:** 2013-11-12

**Authors:** Robert Lenartowski, Anna Suwińska, Justyna Prusińska, Krzysztof Gumowski, Marta Lenartowska

**Affiliations:** 1 Laboratory of Isotope and Instrumental Analysis, Faculty of Biology and Environment Protection, Nicolaus Copernicus University, Toruń, Poland; 2 Laboratory of Developmental Biology, Faculty of Biology and Environment Protection, Nicolaus Copernicus University, Toruń, Poland; 3 School of Life Sciences, University of Warwick, Wellesbourne, UK; 4 Department of Molecular and Cellular Biology, University of Gdańsk, Gdańsk, Poland

**Keywords:** Calcium homeostasis, Calreticulin, Chaperone activity, Gene expression, Nucleolus, Pollen–pistil interactions

## Abstract

Calreticulin (CRT) is a highly conserved and ubiquitously expressed Ca^2+^-binding protein in multicellular eukaryotes. As an endoplasmic reticulum-resident protein, CRT plays a key role in many cellular processes including Ca^2+^ storage and release, protein synthesis, and molecular chaperoning in both animals and plants. CRT has long been suggested to play a role in plant sexual reproduction. To begin to address this possibility, we cloned and characterized the full-length cDNA of a new *CRT* gene (*PhCRT*) from *Petunia*. The deduced amino acid sequence of *Ph*CRT shares homology with other known plant CRTs, and phylogenetic analysis indicates that the *PhCRT* cDNA clone belongs to the CRT1/CRT2 subclass. Northern blot analysis and fluorescent in situ hybridization were used to assess *PhCRT* gene expression in different parts of the pistil before pollination, during subsequent stages of the progamic phase, and at fertilization. The highest level of *PhCRT* mRNA was detected in the stigma–style part of the unpollinated pistil 1 day before anthesis and during the early stage of the progamic phase, when pollen is germinated and tubes outgrow on the stigma. In the ovary, *PhCRT* mRNA was most abundant after pollination and reached maximum at the late stage of the progamic phase, when pollen tubes grow into the ovules and fertilization occurs. *PhCRT* mRNA transcripts were seen to accumulate predominantly in transmitting tract cells of maturing and receptive stigma, in germinated pollen/growing tubes, and at the micropylar region of the ovule, where the female gametophyte is located. From these results, we suggest that *PhCRT* gene expression is up-regulated during secretory activity of the pistil transmitting tract cells, pollen germination and outgrowth of the tubes, and then during gamete fusion and early embryogenesis.

## Introduction

In double fertilization, a reproductive system unique to flowering plants, two immotile sperm cells are delivered to the female gametophyte (the embryo sac) by a pollen tube. The male gametes are released into the embryo sac, usually within one of the two synergid cells commonly known as the receptive synergid. One sperm cell fuses with the egg cell to generate a zygote/embryo, whereas the other fuses with the central cell to form the nutritive endosperm. Ca^2+^ plays essential signaling, physiological, and regulatory roles during this multi-step process, which comprises three successive phases: pollination, progamic phase, and gamete fusion (see review by Ge et al. 2007).

A good candidate protein to facilitate this Ca^2+^ homeostasis is CRT, a key Ca^2+^-binding/buffering ER-resident protein that is highly conserved and extensively expressed in all eukaryotic organisms investigated, with the exception of yeast (see reviews by Jia et al. 2009; Michalak et al. 2009). In animals, CRT is involved in many different intra- and extracellular processes, such as Ca^2+^ storage and signaling, molecular chaperone activity in the ER, regulation of gene expression, control of cell adhesion and migration, immune regulation, apoptosis, and pathogenesis (see reviews by Michalak et al. 2009; Gold et al. 2010; Wang et al. 2012). Plant CRT has the same molecular structure as the animal protein and shares its chaperone and Ca^2+^ binding activities (see reviews by Crofts and Dencke 1998; Mariani et al. 2003; Jia et al. 2009; Thelin et al. 2011; Li and Yang 2012).

Two *CRT* genes (*CRT1* and *CRT2*) exist in the human, mouse, pig, and rat genomes (Persson et al. 2002). The expression profiles of these two mammalian genes have led to the suggestion that CRT1 is the main protein isoform, whereas CRT2 is testis specific. More than 120 plant *CRT* sequences are described in current gene databases, and *CRT* genes have been isolated from barley (Chen et al. 1994), tobacco (Denecke et al. 1995), maize (Kwiatkowski et al. 1995; Dresselhaus et al. 1996), Chinese cabbage (Lim et al. 1996), *Ricinus* (Coughlan et al. 1997), *Arabidopsis* (Nelson et al. 1997), rice (Li and Komatsu 2000), and wheat (Jia et al. 2008; An et al. 2011). In higher plants, the CRT family consists of three members, which are classified into two distinct subclasses: CRT1/CRT2 (also designated CRT1a/CRT1b) and CRT3 (Nelson et al. 1997; Persson et al. 2003; Christensen et al. 2010). Sequence homology of plant CRTs suggests that CRT1 and CRT2 are similar to each other and correspond with the animal CRT1, whereas the plant-specific CRT3 genes are more highly conserved across species (see review by Jia et al. 2009).

*CRT* expression in plants has been correlated with tissue regeneration, immunity, abiotic stress responses, and cell-to-cell communication by plasmodesmata (see reviews by Jia et al. 2009; Thelin et al. 2011). There are also some indications that CRT may be involved in reproductive events in plants. CRT is expressed abundantly in different flower organs (Denecke et al. 1995; Hassan et al. 1995; Coughlan et al. 1997; Nelson et al. 1997; Borisjuk et al. 1998; Navazio et al. 2002; Hsieh and Huang 2005; Nardi et al. 2006; Christensen et al. 2010) and during pollen–pistil interactions, fertilization, embryogenesis, and seed germination (Chen et al. 1994; Denecke et al. 1995; Dresselhaus et al. 1996; Coughlan et al. 1997; Nelson et al. 1997; Williams et al. 1997; Borisjuk et al. 1998; Lenartowska et al. 2001, 2002, 2009; Christensen et al. 2010; Li et al. 2011). *CRT’s* expression pattern suggests that it could play a role in regulation of Ca^2+^ homeostasis during pollen–pistil interactions and thus contribute to successful fertilization. Some reports indicate that plant *CRT* expression is modulated during sexual reproduction, and in some plants a higher level of *CRT* transcripts has been detected in gametes and zygotes than in vegetative cells (Denecke et al. 1995; Dresselhaus et al. 1996; Nelson et al. 1997). Our previous work (Lenartowska et al. 2001, 2009) examined the possibility that *CRT* is expressed during pollination in *Petunia* and *Haemanthus*. However, at the time of those studies, we were limited to examining *CRT* mRNA expression using probes derived from other plants. Here we report cloning of the *Petunia* *CRT* homolog, *PhCRT*. We identify a full-length cDNA sequence and describe its molecular characteristics and phylogenetic relationships to other plant *CRT* genes. We show that *PhCRT* is highly expressed during pistil transmitting tract maturation, pollen germination and tube outgrowth on the stigma, and then during fertilization and early embryogenesis. We discuss the timing of expression and subcellular localization of *PhCRT* transcripts with respect to the possible functions of CRT in plant sexual reproduction.

## Materials and methods

### Plant material

Commercial cultivars of *Petunia hybrida* were grown at room temperature (Laboratory of Developmental Biology, Faculty of Biology and Environment Protection, Nicolaus Copernicus University, Toruń, Poland). Whole *Petunia* pistils were dissected from closed flowers 1 day before anthesis, from unpollinated flowers at anthesis, and from flowers pollinated with compatible pollen at anthesis. To examine pollen tube growth during the progamic phase, pollinated pistils were dissected from the flowers at different time points after pollination, cut along the longitudinal axis, stained with 0.1 % aniline blue according to the standard protocol, and observed by fluorescence microscopy. For Northern blot analysis, whole pistils or pistils divided into stigma–style fragments and ovaries were frozen in liquid nitrogen and stored at −80 °C until they were used. For fluorescent in situ hybridization, samples of unpollinated and pollinated pistils (fragments of stigmas and ovaries) were fixed, dehydrated in ethanol, and embedded as previously described (Lenartowska et al. 2009).

### RNA extraction and RT-PCR analysis

Plant material (100 mg) was ground in liquid nitrogen. Total RNA was extracted in TRI Reagent^®^ (Sigma) according to the manufacturer’s protocol. First-strand *CRT* cDNA synthesis was performed with 1 μg of total RNA, SuperScript III reverse transcriptase, and an oligo(dT)_20_ primer according to the manufacturer’s instruction (Life Technologies). Nested PCR was done to amplify the internal part of the *PhCRT* cDNA. In brief: 2 μl of first-strand cDNA was used as template for PCR amplification with Pfx50™ DNA polymerase and outer gene-specific primers (Table 1). A 1 μl aliquot of the first PCR mixture served as the template in a second PCR using the inner gene-specific primers (Table 1). PCR cycles were as follows: 95 °C for 15 s followed by 35 cycles of 95 °C for 1 min, 57 °C for 1 min, 72 °C for 1 min, followed by a final extension step of 72 °C for 4 min. First-strand *Petunia* *18S* cDNA synthesis was performed as above except that random hexamer oligonucleotides were used in the first PCR reaction.

**Table 1:** List of primers used in gene cloning in the present study

Amplified sequence/type of reaction	Sense primer	Antisense primer
3′ RACE reverse transcription		3′-RACE adapter 5′-GCGAGCACAGAATTAATACGACTCACTATAGGT12VN-3′
3′ *CRT* cDNA end/outer 3′-RACE PCR	3′-RACE gene-specific outer primer (RL035) 5′-GGAAGGCACCTATGATTGACAACC-3′	3′-RACE outer primer 5′-GCGAGCACAGAATTAATACGACT-3′
3′ *CRT* cDNA end/inner 3′-RACE PCR	3′-RACE gene-specific inner primer (RL036) 5′-TTCAAGGATGACCCCGATCTC-3′	3′-RACE inner primer 5′-CGCGGATCCGAATTAATACGACTCACTATAGG-3′
5′ *CRT* cDNA end/outer 5′-RACE PCR	5′-RACE outer primer 5′-GCTGATGGCGATGAATGAACACTG-3′	5′-RACE gene-specific outer primer (RL037) 5′-CCACCACAGTCAAGCTTCTGCT-3′
5′ *CRT* cDNA end/inner 5′-RACE PCR	5′-RACE inner primer 5′-CGCGGATCCGAACACTGCGTTTGCTGGCTTTGATG-3′	5′-RACE gene-specific inner primer (RL038) 5′-GCTGGTCTGGATACCTTTGTCATT-3′
Internal part of *Petunia* *CRT*/first PCR	CRT outer primer (RL039) 5′-GAGGGTGGCAATGGCTACTCAA-3′	*CRT* outer primer (RL040) 5′-TCGGCATCAGAATCAGCTGGA-3′
Internal part of *Petunia* *CRT*/second PCR	Nested gene-specific primer for *CRT* (RL041) 5′-CTGTCGTCGCAGCTGATGTCTT-3′	Nested gene-specific primer for CRT (RL042) 5′-GCTTGGCATATTCTGGATCATCAC-3′
Full-length *Petunia* *CRT* cDNA	RL068 5′-AGTCGTATTTTCCATCACATAGCAGT-3′	RL071 5′-GTGTAATTTCAACACTATTTAATAAAAATCTAGTC-3′
Internal part of *Petunia 18S* rRNA	RL097 5′-GAGCTAATACGTGCAACAAACC-3′	RL098 5′-ACTAGGACGGTATCTGATCGTCT-3′

Sequence of the adapter ligated to treated mRNA 5′-GCUGAUGGCGAUGAAUGAACACUGCGUUUGCUGGCUUUGAUGAAA-3′ in the 5′ RACE reaction

### Random amplification of cDNA ends (RACE)

RACE was carried out using the FirstChoice RLM-RACE kit (Life Technologies) according to the manufacturer’s protocol. For 3′-RACE, gene-specific primers were based on the 982 bp internal sequence of *PhCRT* cloned in the pJET 1.2 vector. Reverse transcription was performed with the M-MLV enzyme and 3′ RACE Adapter (Table 1) in the presence of RNase inhibitor; 1 μl of the reaction was then used in the outer 3′-RACE PCR with the 3′ RACE Outer Primer and 3′ RACE gene-specific outer primer (Table 1), and Pfx50™ DNA polymerase. PCR cycles were as follows: 95 °C for 15 s followed by 35 cycles of 95 °C for 30 s, 57 °C for 1 min, 72 °C for 1 min, followed by a final extension step of 72 °C for 4 min. A 1-μl aliquot of the outer 3′-RACE PCR mixture served as the template in the inner 3′-RACE PCR using 3′-RACE Inner Primer and 3′-RACE gene-specific inner primer (Table 1). PCR cycles were as above.

For 5′ RACE, total RNA was dephosphorylated with calf intestinal phosphatase and treated with tobacco acid pyrophosphatase to remove the 5′ cap structure from the full-length mRNA. 5′-RACE Adapter was ligated to treated mRNA with T4 RNA ligase (see the full-length sequence under the Table 1). Reverse transcription was performed with random decamers in the presence of the M-MLV enzyme and RNase inhibitor at 42 °C for 1 h. The 5′ RACE primers were designed according to the cloned internal fragment sequence of the *PhCRT* cDNA. A 1-μl aliquot of the reverse transcription reaction was used in the outer 5′-RACE PCR in the presence of the 5′-RACE Outer Primer, 5′-RACE gene-specific outer primer and Pfx50™ DNA polymerase (Table 1). PCR cycles were as follow: 95 °C for 15 s followed by 35 cycles of 95 °C for 30 s, 68 °C for 1 min, 72 °C for 1 min, followed by a final extension step of 72 °C for 4 min. A 1-μl aliquot of the outer 5′-RACE PCR mixture served as the template in an inner 5′-RACE PCR using the 5′-RACE Inner primer and 5′ RACE gene-specific inner primer (Table 1). PCR cycles were as above. The 3′ and 5′ RACE PCR products were visually inspected on a 1 % agarose gel in TBE buffer.

### Plasmid construction and molecular probe synthesis

*PhCRT* RT-PCR or RACE products were cloned into the pJET 1.2 vector using the CloneJET™ PCR Cloning Kit (Thermo Scientific) according to the protocol provided with the kit. The insert was verified by sequencing for its correct amplification and orientation in pJET. To prepare the full-length *PhCRT* antisense probe, the plasmid containing the *PhCRT* insert was cut with Xba I and purified on CHROMA SPIN™ + TE − 100 columns. A 1-μg aliquot served as template to generate the digoxigenin (DIG)-labeled probe using the DIG RNA Labeling Kit (SP6/T7) according to the manufacturer’s instructions (Roche). An RT-PCR cDNA containing the internal part of the *Petunia* *18S* rRNA sequence was cloned in pJET 1.2 vector. DIG-labeled *18S* probe was generated by PCR according to the manufacturer’s instructions (PCR DIG Synthesis Kit, Roche) with uncut plasmid DNA as the template (Table 1).

### Northern blot analysis

Total RNA concentrations were measured with a NanoDrop ND-1000 spectrophotometer (NanoDrop Technologies), and RNA quality was assessed by visualization on a 1 % agarose gel in 1× TBE buffer. A 15-μg aliquot of total RNA was dried under a vacuum, resuspended in 5 μl of RNase-free H_2_O, supplemented with two volumes of denaturing buffer containing 50 % formamide, 6.1 % formaldehyde, 1× MOPS, 1× loading dye buffer, and 40 mg/dm^−3^ ethidium bromide, and heat denatured. RNA was resolved on a 1.2 % formaldehyde-agarose gel, capillary transferred onto a nylon membrane (Hybond-N^+^, Amersham) in 20× SSC buffer, rinsed with distilled water, and UV cross-linked in the GS Gene Linker^®^ UV Chamber (BioRad). Membranes were prehybridized for 40 min in DIG Easy Hyb buffer (Roche) at 65 °C, and then hybridized overnight to the DIG-labeled full-length *PhCRT* antisense probe at a final concentration 150 ng/ml. Chemiluminescence detection was performed according to the manufacturer’s instruction (DIG Luminescent Detection Kit, Roche). To reprobe the membrane with *Petunia 18S* DIG-labeled probe, the blot was stripped twice at 80 °C in 50 % formamide, 5 % SDS, 50 mM Tris–HCl, pH 7.5, for 60 min, and then rinsed with 2× SSC. Rehybridization was performed in the same conditions as hybridization described above. Northern hybridization was performed a minimum of three times for each experiment, and representative data are shown. Quantification of signals was done with Image Gauge 3.4 software (Science Lab99). All data obtained from the Northern blot experiments were subjected to one-way ANOVA test.

### Fluorescent in situ hybridization (FISH)

Samples of plant material were fixed with freshly prepared 4 % formaldehyde in phosphate-buffered saline (PBS), pH 7.2, overnight at 4 °C. After fixation, specimens were washed in PBS, dehydrated through a graded series of ethanol, embedded in LR Gold resin (Fluka), sectioned with a diamond knife into semi-thin sections (longitudinal sections of stigmas and ovary fragments), and transferred onto microscope slides covered with Biobond (BioCell). The DIG-labeled antisense *PhCRT* RNA probe generated above was used at a final concentration of 0.5 μg/μl. Pre-hybridization and hybridization were carried out in 50 % formamide, 4× SSC, 5× Denhardt’s, and 50 mM sodium phosphate buffer for 1 h at 42 °C and overnight at 37 °C, respectively. Signals were detected using primary mouse anti-DIG and secondary goat-anti-mouse IgG-Alexa Fluor 488 antibodies (Roche and Life Technologies, respectively). A no-probe control was also performed. In the final step, DNA was stained with 2 μg/ml 4′, 6-diamidino-2-phenylindole (DAPI, Fluka). Images were acquired using an Olympus BX50 fluorescence microscope, Olympus XC50 digital color camera, and CellB software (Olympus Soft Imaging Solutions GmbH).

### In silico sequence analysis

The Neighbor-joining tree estimated from distances between the plant CRT amino acid sequences was built by Mega version 4 http://www.megasoftware.net/, using Tajima-Nei distance and 1000 bootstrap (Tamura et al. 2007). Plant CRTs were aligned in ClustalW2 http://www.ebi.ac.uk/Tools/msa/clustalw2/ (Larkin et al. 2007). *Ph*CRT molecular mass and phosphorylation sites were predicted by ProtParam and NetPhos programs, respectively (Blom et al. 1999; Gasteiger et al. 2005). CRT amino acid sequences from different species were retrieved from GenBank http://www.ncbi.nlm.nih.gov. *Petunia* Expressed Sequence Tags (EST) database sequences showing homology to the *Arabidopsis* *CRT* cDNA clone (NM_001036122) were identified with the BLAST program http://blast.ncbi.nlm.nih.gov/Blast.cgi (Altschul et al. 1990). Primer3Plus was used to design gene-specific primers http://www.bioinformatics.nl/cgi-bin/primer3plus/primer3plus.cgi/ (Untergasser et al. 2007). Statistical analysis was carried out by one-way ANOVA http://www.danielsoper.com/statcalc3/calc.aspx?id=43.

## Results

### *PhCRT* gene cloning and sequence analysis

**Fig. 1:**
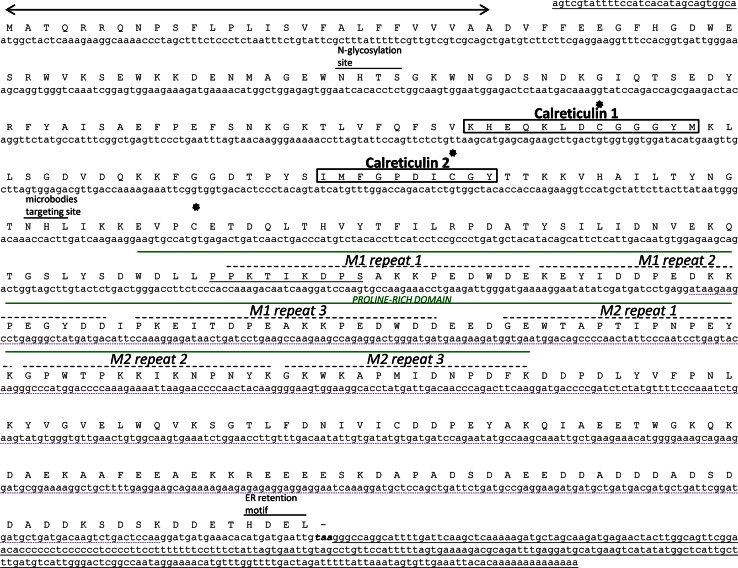
Nucleotide sequence of the *PhCRT* cDNA and its predicted amino acid sequence. The predicted signal peptide is indicated by a *two*-*headed* *arrow*. CRT-family signature motifs 1 and 2 are *boxed*. *Black stars* show locations of three cysteine residues. The proline-rich domain is marked by *solid line* above the amino acid sequence. The putative nuclear targeting sequence (PPKXIKDPX) is *underlined* at the beginning of the P-domain. Triplicate repeats M1 (PXXIXDPXXKKPEXWDE) and M2 (GXWXAXXIXNPXYK) are marked with *dotted lines*. *Short* *solid lines* over the amino acid sequence indicate potential motifs found in the *Ph*CRT sequence. The nucleotide sequences of the 5′ and 3′ UTRs are *underlined* with a *solid line*. The termination codon is indicated in *bold*. The 3′ EST sequence residing in the full-length *PhCRT* cDNA is *underlined* with a *dashed line*

Full-length *Arabidopsis* *CRT* cDNA sequence (NM_001036122) was used to search the *Petunia* EST database. Three *Petunia* EST sequences (CV296977.1, CV296202.1, and DC240877.1) showed homology to the 5′ end of the *Arabidopsis CRT* cDNA, and one (DW177206.1) showed homology to the 3′ end. Because none of the 5′ EST sequences overlapped with the 3′ EST sequence, we designed primers to amplify the *PhCRT* cDNA inner sequence. The obtained 982-bp inner sequence was then used to design gene-specific primers that were used to clone the *PhCRT* 3′ and 5′ ends by rapid amplification of cDNA ends. The resulting fragments were sequenced and used to design a set of primers to amplify a full-length *PhCRT* cDNA, which was cloned into the pJET 1.2 vector. The *PhCRT* cDNA sequence is 1,550 bp and consists of a 30-bp 5′ untranslated region (UTR) upstream of the ATG initiation codon, a 1,254-bp open reading frame (ORF) that terminates with a TAA stop codon, and a 266-bp 3′ UTR (Fig. 1). Two putative polyadenylation signal sequences (ATTAAA and TTAAAT) (Nevins 1983) were mapped at the 3′-end of the *PhCRT* sequence, 17 and 16 bp upstream of the poly(A) tail, respectively. (The full-lenght *PhCRT* cDNA sequence was submitted to the European Nucleotide Archive (accession number HG738129)).

### Deduced amino acid sequence analysis of the *PhCRT* cDNA clone

The putative *PhCRT* ORF encodes a relatively acidic (pI 4.43, as predicted by ProtParam software (Gasteiger et al. 2005) 417-amino acid polypeptide with a calculated molecular mass of 47.77 kDa. The predicted molecular mass is smaller than that of native CRT isolated from *Petunia*, which migrates at 58 kDa on an SDS-PAGE gel (data not shown). The NetPhos program (Blom et al. 1999) predicts that *Ph*CRT has eight to ten putative phosphorylation sites for serine, tyrosine, or threonine kinases. Similar to other plant CRTs, *Ph*CRT has three evolutionarily conserved domains: N, P and C. The globular N-domain starts with a predicted signal peptide and contains an *N*-glycosylation consensus sequence (NHTS) at amino acids 59–62 and a microbodies targeting site at amino acids 162–164 (Fig. 1). The location of the *N*-glycosylation site is species-specific within the plant CRT family (see review by Jia et al. 2009). A search of structural motifs revealed two conserved CRT-family signature regions, calreticulin 1 and calreticulin 2, in the N-domain (Fig. 1). Three cysteine residues were found in this region (Fig. 1); the second and third cysteine residues could form a disulphide bridge that is essential for proper folding of the N-terminal region (see review by Jia et al. 2009). The N-domain is also enriched for histidine residues, which most likely mediate Zn^2+^ binding (see review by Michalak et al. 2009). A highly conserved proline-rich region, the P-domain, extends from amino acid 208 to amino acid 309. This region contains three repeats each of two proline-rich sequences, M1 (PXXIXDPXXKKPEXWDE) and M2 (GXWXAXXIXNPXYK) (Fig. 1). Both motifs contain a high percentage of acidic amino acids, which are required for low capacity, but relatively high-affinity Ca^2+^-binding; this region is responsible for the CRT lectin-like chaperone activity (see review by Michalak et al. 2009). The C-terminus of CRT is enriched in negatively charged residues that are responsible for binding Ca^2+^ with relatively high capacity, but low affinity. This domain ends with an HDEL sequence, which is required for targeting and retention of CRT in the ER lumen (see review by Jia et al. 2009).

**Fig. 2:**
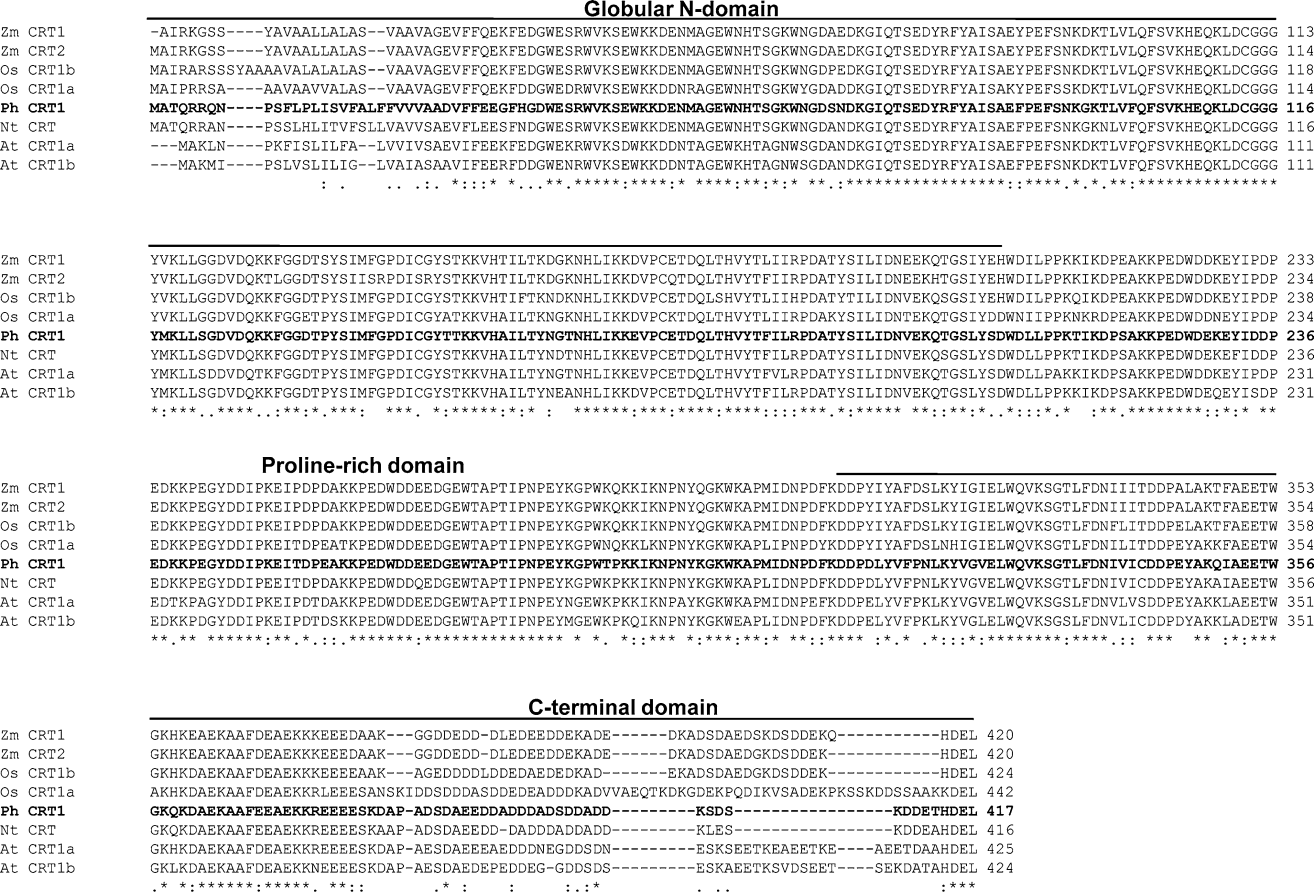
Sequence alignment of *Ph*CRT with other known plant CRTs: *Arabidopsis* (At CRT1a, At CRT1b), *Nicotiana* (Nt CRT), *Oryza* (Os CRT1a, Os CRT1b), and *Zea* (Zm CRT1, Zm CRT2). *Ph*CRT amino acid sequence is given in *bold*. Sequence data were obtained from GenBank with the following accession nos.: *Arabidopsis thaliana* CRT1a (AAC49695), CRT1b (AEE28414); *Nicotiana tabacum* (ACH72686); *Oryza sativa* CRT1a (BAF13713), CRT1b (BAF21197); *Zea mays* CRT1 (CAA86728) and CRT2 (AAF01470). *Petunia hybrida* CRT1 (HG738129) was deposited in the European Nucleotide Archive. The globular N-domain and C-terminal domain are indicated by lines under the amino acid sequence. The numbers on the right indicate the amino acid position. *Asterisks* denote positions that have a single, fully conserved residue. *Colon* or *dot* indicates conservation between groups of strongly similar properties (>0.5 in the Gonnet PAM 250 matrix) or weakly similar properties (<0.5 in the Gonnet PAM 250 matrix), respectively (Larkin et al. 2007)

**Fig. 3:**
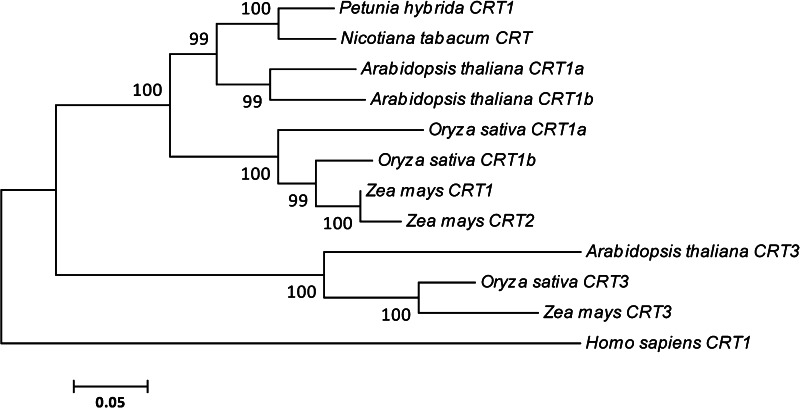
A rooted neighbor-joining phylogram of plant CRTs. The evolutionary history was inferred using the Neighbor-Joining method (Saitou and Nei 1987). The bootstrap consensus tree inferred from 1,000 replicates is taken to represent the evolutionary history of the taxa analyzed (Felsenstein 1985). Branches corresponding to partitions reproduced in less than 50 % of the bootstrap replicates are collapsed. Bootstrap values out of 1,000 are indicated on the nodes of the tree (Felsenstein 1985). The tree is drawn to scale, with branch lengths in the same units as those of the evolutionary distances used to infer the phylogenetic tree. The evolutionary distances were computed using the Poisson correction method (Zuckerkandl and Pauling 1965) and are in the units of the number of amino acid substitutions per site. All positions containing gaps and missing data were eliminated from the dataset (Complete deletion option). Phylogenetic analyses were conducted in MEGA4 (Tamura et al. 2007). Human CRT1 was used as the outgroup. Two distinct clusters can be observed: CRT1 (CRT1a) and CRT2 (CRT1b) isoforms vs. CRT3 isoforms. The GenBank accession number for *Homo sapiens* CRT1 is AAA51916; *Arabidopsis thaliana* CRT3 (NP_563816); *Oryza sativa* CRT3 (BAC06263); *Zea mays* CRT3 (ACG33961); all others are the same numbers as shown in Fig. 2

The *Ph*CRT sequence was compared to several of the full-length CRT1/CRT2 sequences found in the GenBank database. As shown Fig. 2, the ClustalW2 program revealed that the deduced amino acid sequence of *Ph*CRT shared high identity with CRTs from *Nicotiana* (91.83 %), *Arabidopsis* CRT1a and CRT1b (80.1 and 76.74 %, respectively), *Oryza* CRT1a and CRT1b (71.22 and 74.1 %, respectively), and maize CRT1 and CRT2 (74.58 and 72.66 %, respectively). We used MEGA 4.1 and the neighbor-joining method to generate a phylogenic tree of the plant CRT amino acid sequences. Human CRT, which shares 48.4 % identity with *Petunia* CRT (Fig. 2) was used as an outgroup. The resulting tree (Fig. 3), groups the eleven plant CRTs into two large clusters of CRT1a/b (or CRT1/CRT2) and CRT3. Cluster analysis suggests that the *PhCRT* gene belongs to the CRT1/CRT2 group (Fig. 3). The CRT1/CRT2 group contains the CRTs that play general chaperone functions in plants (Christensen et al. 2010).

### Expression pattern of the *PhCRT* gene before pollination and during the progamic phase

**Fig. 4:**
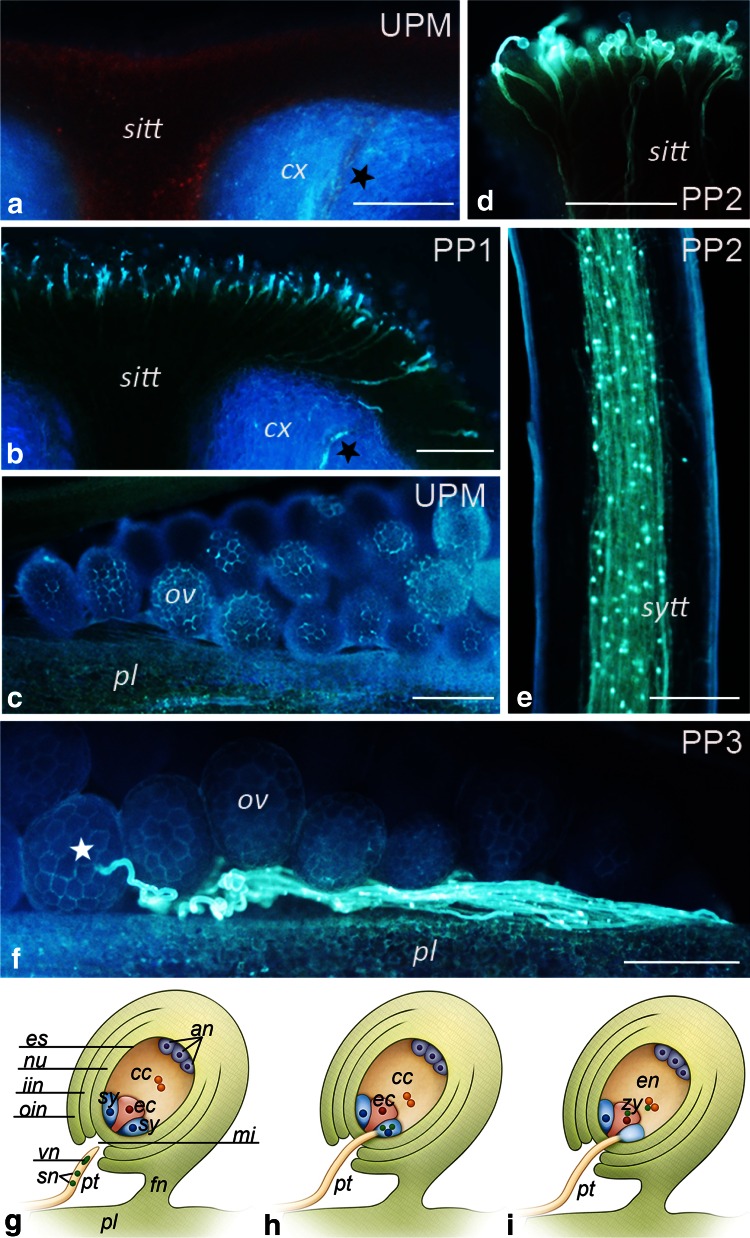
Aniline *blue staining* (glaucous fluorescence) of germinated pollen and pollen tubes growing in planta (**a**–**f**) and schematic representations of mature *Petunia* ovules before and at fertilization (**g**–**i**). All pictures from **a** to **f** show longitudinal sections of stigma, style, or ovary fragments. **a** Stigma of UPM pistil; autofluorescence (*red*) of starch in amyloplasts is visible in the stigma transmitting tract (*sitt*). **b** Stigma of pollinated pistil at the PP1 stage; germinated pollen and outgrowing tubes are visible in the *sitt*. **c** Ovary of UPM pistil; similar images showing lack of pollen tubes were observed in pollinated pistils at the PP1 and PP2 stages (data not shown). Stigma (**d**) and style (**e**) at the PP2 stage; whole *sitt* and style transmitting tract (*sytt*) are penetrated by pollen tubes; some callose plugs (*bright points*) are visible in long tubes (**e**). **f** Ovary of pollinated pistil at the PP3 stage; pollen tubes growing on the placenta (*pl*) and ingrowing into ovules (*ov*) are visible at this stage. *Black stars* on **a** and **b** show vascular strand, *white star* on **f** shows pollen tube ingrowth into an ovule. **g** Unfertilized ovule at the PP3 stage; the embryo sac contains the egg apparatus, which consists of two synergids (*sy*), the egg cell (*ec*), the central cell (*cc*), and three antipodals (*an*). **h** Ovule at the end of the PP3 stage (moment of sperm cells release into the receptive synergid). **i** Fertilized ovule with the zygote (*zy*) and endosperm (*en*); the receptive synergid is degenerated. *cx* cortex, *es* embryo sac, *fn* funicle, *iin* inner integument, *mi* micropyle, *nu* nucellus, *oin* outer integument, *pt* pollen tube, *sn* sperm nuclei, *vn* vegetative nucleus. *Bars* 100 μm

The *Petunia* pistil is composed of a solid style filled with transmitting tissue and a wet stigma that is covered with exudate at the receptive stage. As shown in Fig. 4, three main stages of the progamic phase have been defined: a first stage, when pollen germinates and tubes outgrow on the stigma (PP1, Fig. 4b); a second stage, when pollen tubes penetrate the stigma/style transmitting tract (PP2, Fig. 4d, e); and a third stage, when pollen tubes grow into the ovules (PP3, Fig. 4f). Figure 4a and c shows fragments of the stigma and the ovary, respectively, of unpollinated mature pistils at anthesis (UPM pistil with exudate on the stigma). The embryo sac (female gametophyte) of *Petunia* develops according to the Polygonum-type (Willemse and Van Went 1984) and consists of two synergids, the egg cell, the central cell, and three antipodals (Van Went and Kwee 1990). A schematic representation of the *Petunia* ovule during the PP3 stage and at fertilization is shown in Fig. 4g–i.

**Fig. 5:**
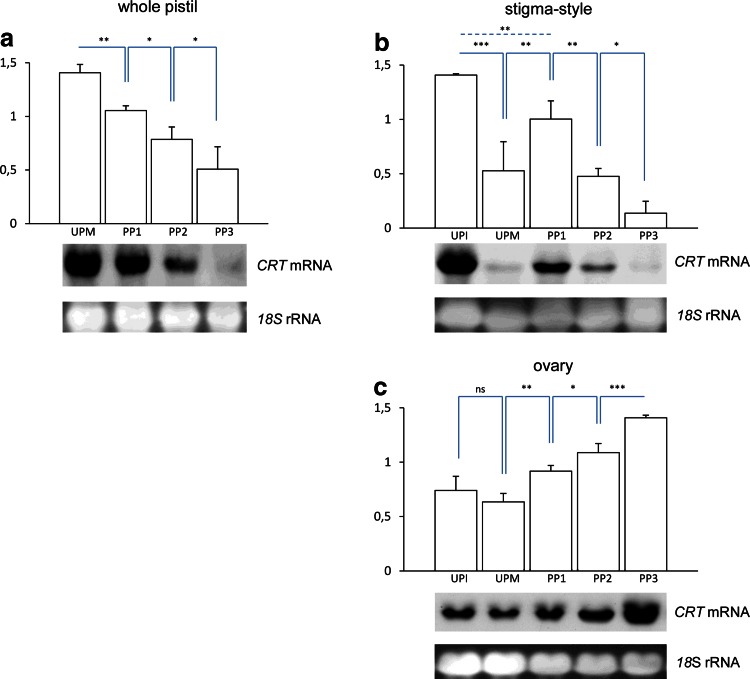
Northern blot analysis of *CRT* mRNA transcript levels in whole *Petunia* pistils (**a**), stigma–style fragments (**b**), and ovaries (**c**) before pollination and during subsequent stages of the progamic phase. *Bars* show the relative *CRT* mRNA levels (mean of three replicates and standard deviation) normalized to the *Petunia* *18S* rRNA levels. Statistical analysis was carried out by one-way ANOVA (*P ≤ 0.05; **P ≤ 0.01; ***P ≤ 0.001). Representative Northern blots are shown under the graphs. Blots were hybridized with *PhCRT* antisense probes, and then re-hybridized with *Petunia* *18S* rRNA probe. *UPI* unpollinated immature pistil, *UPM* unpollinated mature pistil, *PP1*, *PP2,* and *PP3* subsequent stages of the progamic phase (described in detail in “Results”)

To analyze the expression pattern of *PhCRT* in relation to anthesis, pollination, and pollen tube growth from the stigma to the ovary, we first investigated *PhCRT* mRNA transcript levels in whole pistils at 1 day before anthesis (unpollinated immature pistil without exudate on the stigma, UPI), at anthesis (UPM), and during the PP1, PP2, and PP3 stages after pollination. Northern blot analysis of total *Petunia* pistil RNA revealed that the highest level of *PhCRT* expression was at the UPM stage. Expression then decreased gradually through the PP1, PP2, and PP3 stages (Fig. 5a). This result was somewhat surprising because some previous studies in other plant species reported that the highest levels of *CRT* mRNA transcripts were correlated with fertilization, embryogenesis, and seed development (Chen et al. 1994; Nelson et al. 1997; Borisjuk et al. 1998; Navazio et al. 2002). However, most other studies have been carried out on ovaries, and the transcriptional activity of *CRT* genes in whole pistils before pollination and during subsequent stages of the progamic phase has never been examined. Therefore, we compared *PhCRT* mRNA transcript levels in unpollinated pistils before and at anthesis (UPI and UPM pistils, respectively). In addition, to assess developmental and tissue-specific expression of *PhCRT*, we divided the pistils into stigma–style fragments (Fig. 5b) and ovaries (Fig. 5c). Whereas comparable levels of *PhCRT* mRNA transcripts were observed in the ovaries dissected from UPI and UPM pistils (Fig. 5c), levels of *PhCRT* mRNA were about threefold higher in stigma–style fragments of UPI pistils than in the same fragments of UPM pistils (Fig. 5b). These results suggest that elevated expression of *PhCRT* before pollination correlates with maturation of the stigma–style transmitting tract.

Next, we wished to determine whether the expression profiles of *PhCRT* vary between different parts of the pistil before pollination and during subsequent stages of the progamic phase. In the upper part of the pistil (stigma–style fragment, Fig. 5b), the level of *PhCRT* mRNA was significantly higher in the first stage of the progamic phase (PP1) than in the UPM stage, suggesting that pollen germination and tube outgrowth on the stigma induce expression of *PhCRT* or stabilization of the transcript*. PhCRT* mRNA levels dropped at PP2, and PP3, when the entire stigma–style transmitting tract is penetrated by growing pollen tubes. By contrast, in the ovaries, a progressive increase of *PhCRT* mRNA level was observed after pollination and through the progamic phase. The *PhCRT* mRNA level was highest at PP3, when pollen tube ingrowth into the ovule and fertilization is highly probable (Fig. 5c). These results indicate that *PhCRT* transcriptional activity or mRNA stabilization in ovaries might be stimulated by progressive growth of the pollen tubes in the pistil. Moreover, the highest level of *PhCRT* mRNA transcripts in the ovary at the PP3 stage could be indicative of a response to fertilization.

### Subcellular localization of the *PhCRT* mRNA transcripts in stigmas and ovaries before and after pollination

**Fig. 6:**
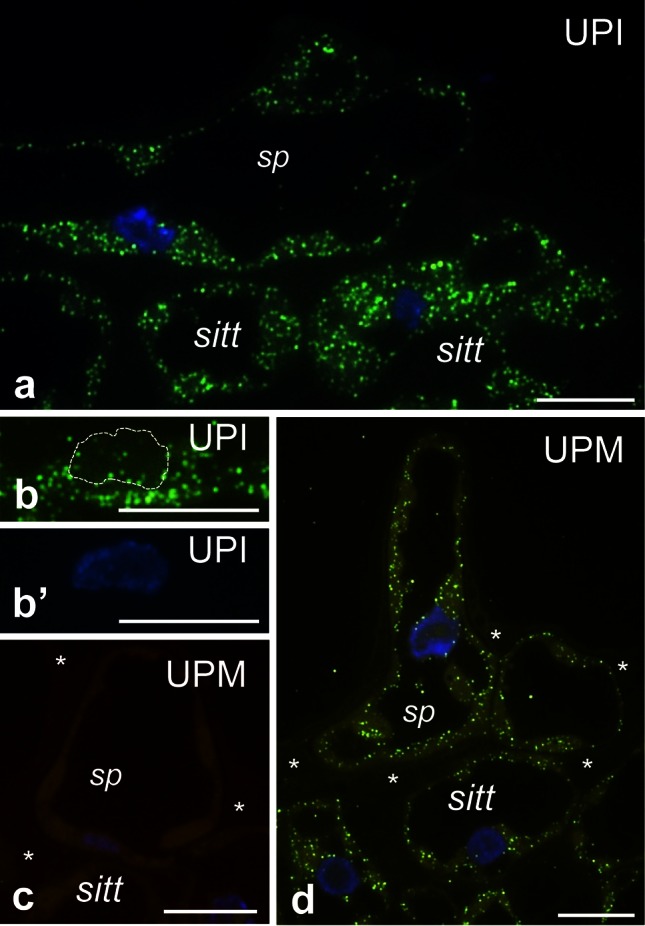
Localization of *PhCRT* mRNA transcripts (*green*) in the stigmas of UPI (**a**–**b’**) and UPM (**d**) pistils. **b** Hybridization signals in *sitt* cell nucleus marked with *dotted line.* **c** No-probe control. Nuclei are stained with DAPI (*blue*). *sitt* stigma transmitting tract cell, *sp* stigma papillae; *stars* in **c, d** shows stigma exudates. *Bars* 10 μm

To determine the spatial distribution of *PhCRT* mRNA transcripts in the somatic cells of the stigma transmitting tract (sitt), germinated pollen, growing pollen tubes, and various cells of the ovaries (somatic cells of placenta and ovule and female gametophyte cells), samples of stigmas and ovaries dissected from unpollinated and pollinated pistils were fixed, embedded, sectioned, and processed for FISH and visualized by fluorescence microscopy before pollination and at the beginning and end of the progamic phase. Longitudinal sections of stigmas dissected from UPI pistils showed accumulation of *PhCRT* mRNA transcripts in the cytoplasm of the sitt secretory cells (Fig. 6a). In those cells, mRNAs were also found to be abundant in the nuclei (Fig. 6b, b’). After anthesis, when stigma exudate was intensively secreted (Fig. 6d, stars), *PhCRT* mRNA transcripts were observed in the cytoplasm of sitt cells (Fig. 6d). However, at this stage (UPM pistil) the level of fluorescence was lower than observed at the UPI stage, and signal was occasionally observed in close proximity to the sitt cell nuclei (Fig. 6d). No labeling was observed when the probe was omitted (Fig. 6c).

**Fig. 7:**
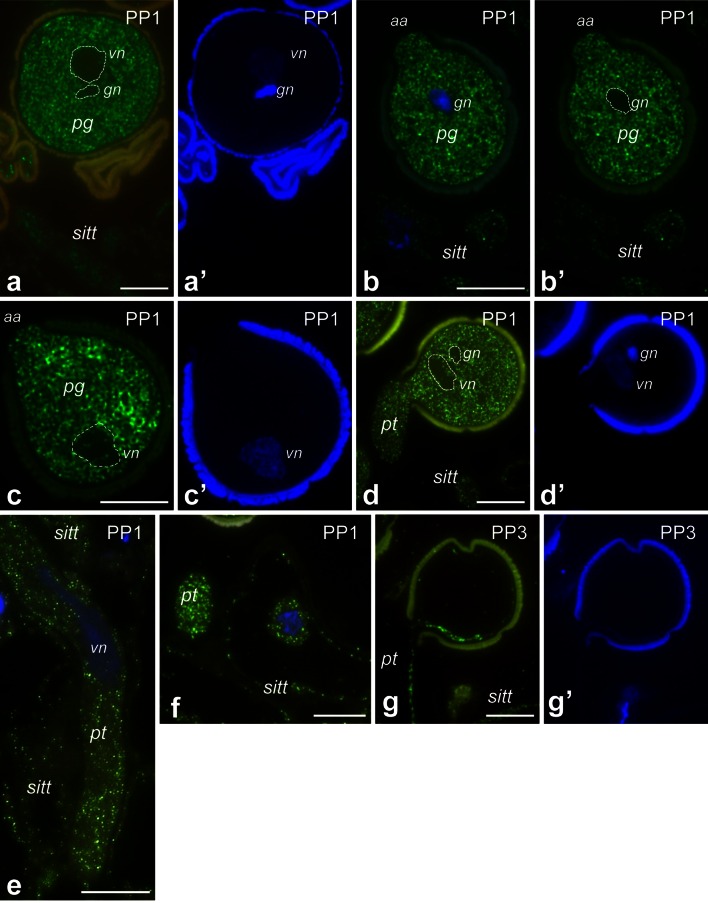
Localization of *PhCRT* mRNA (*green*) in stigmas of pollinated pistils at PP1 (**a**–**f**) and PP3 (**g**) stages. **a**, **a’** Pollen hydrated on the stigma. **b**–**c’** Early germinated pollen. **d**, **d’** Germinating pollen grain with a very short tube. **e** Longitudinal section of a pollen tube elongated on the stigma. **f** Cross-section of the sub-apical zone of a pollen tube growing between sitt cells. **g**, **g’** Vacuolated pollen grain/tube on the stigma. Positions of *sitt* cell nuclei and generative/vegetative nuclei of the male gametophyte (pollen grain/tube) are stained with DAPI (*blue* in **a’**, **b**, **c’**, **d’**, **e**, **f**, **g’**). *aa* active aperture, *gn* generative nucleus, *pg* pollen grain, *pt* pollen tube, *sitt* stigma transmitting tract cell, *vn* vegetative nucleus. *Bars* 10 μm

After pollination, the strongest hybridization signals were observed at the PP1 stage in hydrated (Fig. 7a, a’) and germinated (Fig. 7b, c’) pollen as well as in outgrowing pollen tubes (Fig. 7d, d’). Pollen hydrated and germinated on the stigma surface showed uniform fluorescence throughout the cytoplasm and associated with the active aperture region of germinating pollen (Fig. 7b, b’). As shown in Fig. 7d, e, at the PP1 stage, pollen tubes of various lengths were visible on the stigma sections. In short tubes, *PhCRT* mRNA transcripts were almost uniformly distributed in the cytoplasm with the exception of an extra-apical zone of the tip-growing cell, where signal was weaker (Fig. 7d). In elongated tubes, hybridization signals were detected predominantly along the cytoplasm of the tip-growing cells, and only small regions (probably vacuoles) were devoid of fluorescence (Fig. 7e). In images showing cross-sections of growing pollen tubes, mRNA enrichment was observed at the sub-apical region, where no vacuoles are present (Fig. 7f). At the PP1 stage, sitt cells showed weaker labeling than they did before pollination (compare Fig. 7f with Fig. 6a, d). In these cells, *PhCRT* mRNA transcripts were localized in peripheral cytoplasm and in close proximity to the cell nucleus (Fig. 7f). At the PP3 stage, hybridization signals were only detected in residual cytoplasm along the edge of the vacuolated pollen grains/tubes and crushed sitt cells (Fig. 7g). These results are consistent with our Northern blot analysis and confirm that the highest level of *PhCRT* mRNA accumulation correlates with sitt maturation (which results in the presence of exudates on the receptive stigma), and with pollen germination and pollen tube outgrowth in the stigma.

To gain insight into possible differential transcriptional activity of *PhCRT,* we paid special attention to FISH signals in the nuclei, as these may indicate nascent transcription. Hybridization signals were never detected in generative and vegetative nuclei of hydrated pollen (Fig. 7a, a’). By contrast, label was observed in vegetative nuclei in early germinating pollen (Fig. 7c, c’) and in pollen with very short tubes (Fig. 7d, d’). No signal was detected in vegetative nuclei of elongated pollen tubes (Fig. 7e).

**Fig. 8:**
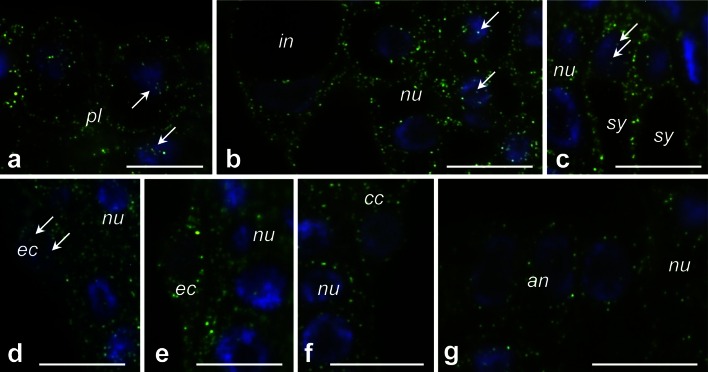
Localization of *PhCRT* mRNA (*green*) in ovaries before pollination. **a** Sporophyte cells of the placenta surface close to the micropylar region of the ovule. **b** Somatic cells of the ovule. **c**–**f** Micropylar end of the embryo sac: sister synergids (**c**), egg cell (**d**, **e**, serial sections of the same egg cell), central cell (**f**). **g** Chalazal end of the embryo sac with antipodals (*an*). Nuclei are stained with DAPI (*blue*). *Arrows* in **a**–**d** show hybridization signals in the nuclei of placenta (*pl*), nucellus (*nu*), synergids (*sy*), and egg cell (*ec*), respectively. *in* integument cell. *Bars* 10 μm

We next examined *PhCRT* FISH signals in longitudinal sections of the ovary before pollination (Fig. 8). At this stage, somatic cells of the placenta and the ovule (nucellus and integuments cells) exhibited diffuse signal throughout the cytoplasm (Fig. 8a, b, respectively). The transcripts were also found in the nuclei of these cells (Fig. 8a, b, arrows). Similar localization patterns were observed in the cytoplasm of the female gametophyte cells, particularly at the micropylar end of the embryo sac. Within each embryo sac, both synergids (Fig. 8c), the egg cell (Fig. 8d, e), and the central cell (Fig. 8f) were all positive for *PhCRT* mRNA transcripts. By contrast, antipodals localized on the chalazal end of the embryo sac showed weaker labeling in the cytoplasm (Fig. 8g). Before pollination, hybridization signals were associated only with the nuclei of the egg apparatus cells (Fig. 8c, d, arrows), and no labeling was detected in the nuclei of the central and antipodal cells.

**Fig. 9:**
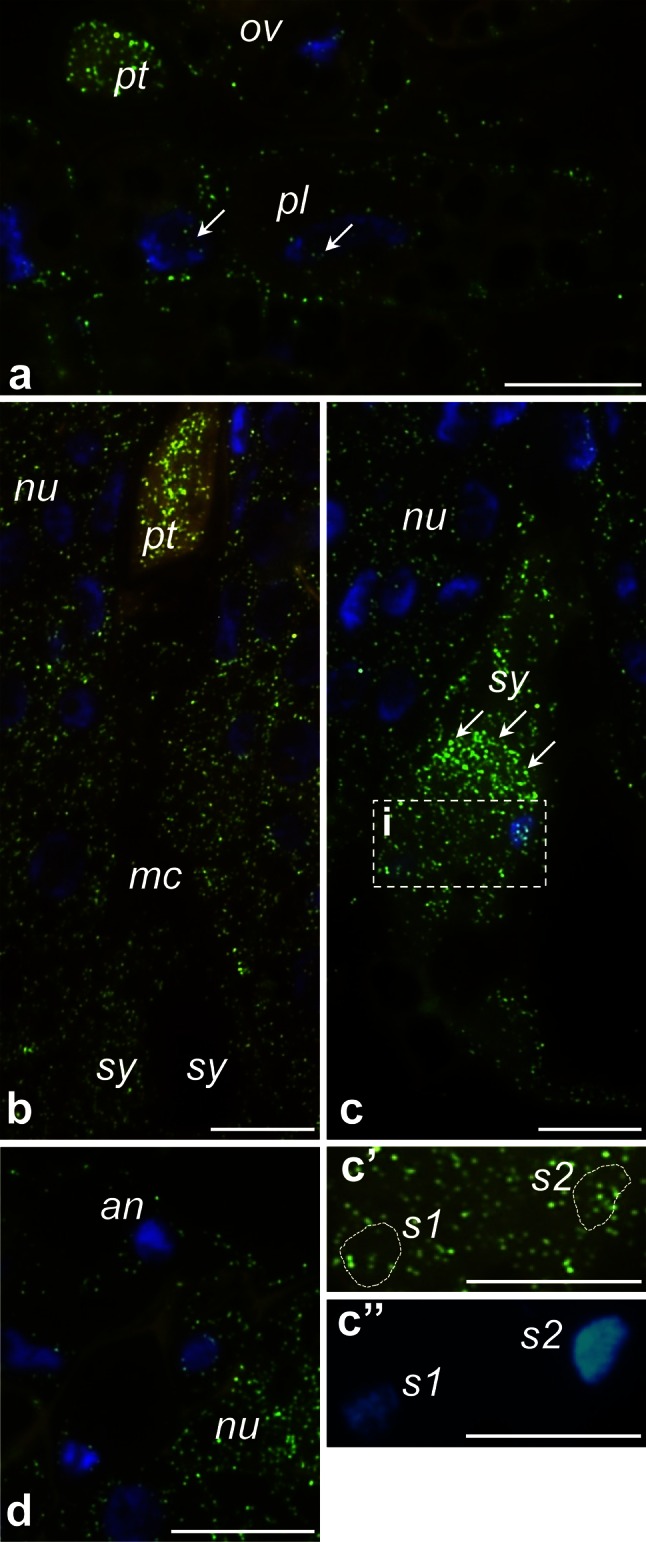
Localization of *PhCRT* mRNA (*green*) in ovaries just before gamete fusion. **a** Sporophytic cells of the placenta (*pl*) surface close to the micropylar region of the ovule (*ov*) with the pollen tube (*pt*). **b** Micropylar canal (*mc*) penetrated by the *pt*. **c** Micropylar region of the embryo sac with receptive synergid (*sy*) at the moment of sperm cells release. FISH and DAPI staining presented in **c’** and **c”** are the higher magnifications of the *boxed region* (i) in **c** showing two sperm cells (*s1* and *s2*) within the receptive synergid. **d** Chalazal region of the embryo sac with antipodal cell (*an*). *Arrows* in **a** show labeling in the nuclei (DAPI-stained, *blue*) of the placenta cells, *arrows* in **c** show *PhCRT* mRNA transcript gradient in the cytoplasm of the receptive synergid. *nu* nucellus cell. *Bars* 10 μm

**Fig. 10:**
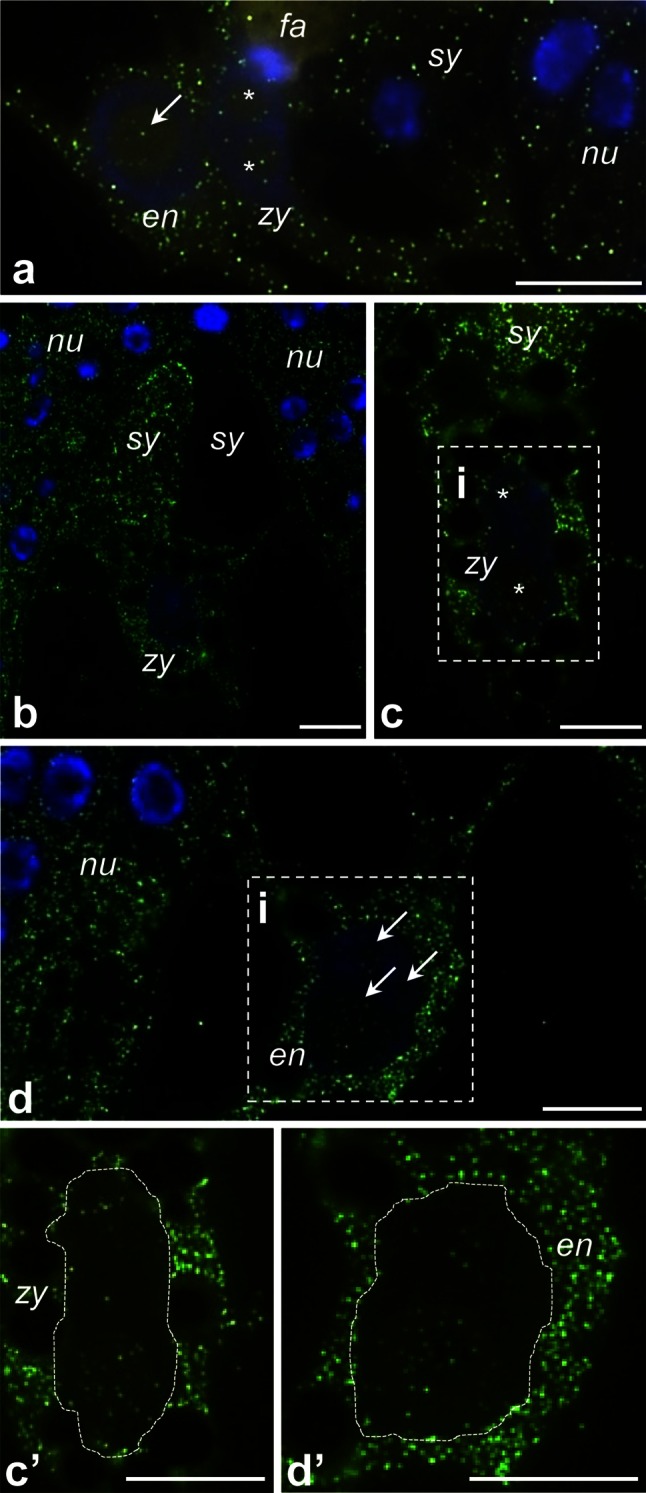
Localization of *PhCRT* mRNA (*green*) in ovaries during fertilization and early embryogenesis. **a** Micropylar region of the embryo sac with the zygote (*zy*), endosperm cell (*en*), and degenerating synergid (*sy*). **b**, **c** Micropylar region of the embryo sac with both synergids and zygote. **d** Micropylar region of the embryo sac with the endosperm cells. **c’**, **d’** are higher magnifications of the *boxed regions* (i) in **c**, **d**, respectively. *Stars* in **a**, **c** and *arrows* in **a**, **d** show nucleolus of the zygote and endosperm cells, respectively. *fa* filiform apparatus of the synergid, *nu* nucellus cell. *Bars* 10 μm

Detailed observations of the ovary samples dissected from pollinated pistils at the PP3 stage revealed that *PhCRT* mRNA transcripts were enriched at the micropylar region of the ovule, where the female gametophyte is located and fertilization occurs inside the embryo sac (Fig. 9, 10). Just before gamete fusion, the strongest signals were detected in pollen tubes, which penetrated the placenta surface (Fig. 9a), and in the micropylar canal of the ovule (Fig. 9b). Preferential accumulation of *PhCRT* mRNA transcripts was also observed in the cytoplasm of the receptive synergid just before gamete fusion (Fig. 9c), when two sperm cells were released (Fig. 9c, boxed region), and during fertilization (Fig. 10b). A *PhCRT* mRNA transcript gradient was visible in the cytoplasm of the receptive synergid at the moment of sperm cells release, at which point signal accumulated in the micropylar pole of the cell (Fig. 9c, arrows). Labeling was also detected in both sperm cells (Fig. 9c’, c’’). During fertilization, high levels of *PhCRT* mRNA transcripts were observed in the zygote (Fig. 10a–c) and endosperm cells (Fig. 10a, d). In these cells, hybridization signals were detected not only throughout the cytoplasm, but also in nuclei (Fig. 10a) and nucleoli of the zygote (Fig. 10a, c, stars and c’, dotted line) and endosperm cells (Fig. 10a, d, arrows and d’, dotted line). At the PP3 stage, high labeling was seen in the cytoplasm of the nucellus cells within both the micropylar and chalazal regions of the ovule (Figs. 10d, 9d, respectively), but very little signal was detected in degenerating synergid (Fig. 10a) or antipodal cells (Fig. 9d). These results are consistent with our Northern blot analysis and confirm that the highest accumulation of *PhCRT* mRNA in the ovary correlates with the late stage of the progamic phase, when pollen tube grows into a receptive synergid, and with gamete fusion and early embryogenesis.

## Discussion

### *PhCRT* is a highly conserved gene belonging to the CRT1/CRT2 subfamily

Here we report the first cloning of a *CRT* gene, *PhCRT*, from the flowering plant *Petunia*. Alignment of the deduced amino acid sequence indicates that *Ph*CRT shares homology with other plant CRTs, from 71 to 91 % identity. *Ph*CRT appears to be most similar to *Nicotiana* CRT and *Arabidopsis* CRT1a and CRT1b isoforms, whereas the similarity between *Ph*CRT and human CRT is lower, only about 48 %. Such low identity level has also been observed between mammalian CRT and other plant CRTs (Li and Komatsu 2000; Jia et al. 2008). Although multiple plant CRT isoforms exist, several regions in their sequences are conserved. Our in silico analysis of the deduced *Ph*CRT amino acid sequence revealed that it has all the typical domains and motifs of a CRT: an N region that contains an ER-targeting sequence, an NHTS consensus sequence, calreticulin 1 and calreticulin 2 motifs, three cysteine residues, a microbodies targeting site, and six histidine residues; a P-domain containing three repeats each of the proline-rich sequences M1 and M2, and a putative nuclear targeting sequence; and a C domain containing an HDEL motif.

Generally, plants contain three CRT isoforms: CRT1 and CRT2 (in *Arabidopsis* CRT1a and CRT1b) represent one subgroup, and CRT3 is a divergent member (see reviews by Jia et al. 2009; Thelin et al. 2011). Some recent reports revealed that the CRT1 and CRT2 family members are components of a general ER chaperone network, whereas the *CRT3* gene is co-expressed with pathogen- and signal transduction-related genes, suggesting functional specialization (Jin et al. 2009; Li et al. 2009; Saijo et al. 2009; Christensen et al. 2010). Our phylogenetic analysis indicates that the *PhCRT* cDNA clone that we identified belongs to the CRT1/CRT2 subclass. This is consistent with the presence of an *N*-glycosylation site at amino acids 59–62. Location of this site near position 50–60 in the N-domain is shared among some other plant CRT1/CRT2 isoforms; in CRT3, the *N*-glycosylation site is usually closer to amino acid 96 (see review by Jia et al. 2009). Moreover, it is thought that tissue-dependent expression differs among members of the *CRT* family. Whereas CRT1 and CRT2 are most abundant in floral, root, and leaf tissues in both *Arabidopsis* and maize, *CRT3* shows highest expression in leaves and roots, but not in flowers. Given the sequence features and our observations that the *PhCRT* gene that we identified is expressed in the pistil, we conclude that it encodes a CRT1/CRT2 homolog and most likely plays general roles associated with protein folding and Ca^2+^ homeostasis.

### The highest expression of *PhCRT* in the ovary correlates with pollen tube entry into the embryo sac, fertilization, and early embryogenesis

We observed high levels of *PhCRT* mRNA in the micropylar region of the ovule before and after pollination and dramatic up-regulation of *PhCRT* expression at the PP3 stage, when transcript accumulation was observed in receptive synergids, and then in zygotes and early endosperms. Hybridization signals were detected in both the cytoplasm and nuclei of these cells, suggesting high transcriptional activity. We did not observe such dramatic increase in *PhCRT* expression level at the PP3 stage in somatic cells of the placenta and ovule, nor in the antipodal cells, which do not participate in double fertilization.

Our Northern blot and FISH data indicated that the maximum level of *PhCRT* expression in the ovary is at the late stage of the progamic phase, when the pollen tube has released sperm cells into the receptive synergid and fertilization has occurred. These results are consistent with findings in several other plant species. Elevated levels of *CRT* mRNA transcripts were observed at the time of fertilization in whole barley ovaries (Chen et al. 1994) in maize zygotes and immature embryos after in vitro fertilization (Dresselhaus et al. 1996), and during somatic and zygotic embryogenesis in *Nicotiana* (Borisjuk et al. 1998). Although, the function of CRT during gamete fusion and embryo development remains unclear, our detailed in situ studies on *CRT* transcriptional activity in the embryo sac during pollen tube entry and early embryogenesis suggest three likely possibilities. One explanation is that enhanced expression of *PhCRT* after fertilization is required for rapid cell divisions of the zygote and early endosperm, as was suggested by Chen et al. (1994) and Dresselhaus et al. (1996). Alternatively, Borisjuk et al. (1998) postulated that the increased *CRT* mRNA level during *Nicotiana* embryogenesis indicates that the chaperone function of CRT is required for proper embryo development and endosperm function. CRT, which is mainly localized in the ER and Golgi, may be required for the proper folding of proteins that are synthesized on the ER and then transported to the Golgi for sorting to their final destinations. Finally, given that precise regulation of Ca^2+^ is required for gamete fusion and activation of the fertilized egg cell (see review by Ge et al. 2007), CRT may be required to maintain Ca^2+^ homeostasis.

We report the first evidence of *PhCRT* mRNA localization in the nucleoli of the zygote and endosperm. In addition to its well-known function in ribosome biogenesis, the plant nucleolus is thought to function in mRNA biogenesis (see review by Shaw and Brown 2011). mRNAs from a wide range of genes were identified in a cDNA library from purified *Arabidopsis* nucleoli. Several hypotheses have been put forth to explain this phenomenon. First, aberrantly spliced mRNAs might localize to the nucleolus for degradation. This could be particularly important in extremely active cells such as those of the early embryo and endosperm. Second, specific mRNAs could have a nucleolar phase preceding their export from the nucleus. Finally, the presence of small regulatory RNAs in the nucleolus suggests that this structure may be involved in regulation of gene expression, possibly in response to cellular conditions (see review by Shaw and Brown 2011). The examined early division stage of *Petunia* embryogenesis represent cell stages with a gene expression program that results in high metabolic activities for cell proliferation and biosynthesis.

### A micropylar-chalazal gradient of *PhCRT* in the receptive synergid

One of our most striking, and novel, observations was that *PhCRT* mRNA formed a gradient in the receptive synergid cell at the PP3 stage. The highest concentration of *PhCRT* mRNA was at the micropylar pole of the cell, above the site of released sperm cells. One possibility is that this expression pattern reflects a role for *PhCRT* in functional differentiation of sister synergids. In all flowering plants examined (except *Plumbago*, which lacks synergids), the highest level of Ca^2+^ in the embryo sac is in the receptive synergid, where Ca^2+^ is also distributed in a micropylar-chalazal gradient (see review by Ge et al. 2007). Generally, only one pollen tube enters each embryo sac in angiosperms, and precise regulation of the Ca^2+^ level at the micropylar region of the penetrated ovule determines its receptivity. Recently, Iwano et al. (2012) showed in *Arabidopsis* that, upon pollen tube arrival at the synergid, Ca^2+^ oscillation begins at the micropylar pole and spreads toward the chalazal pole. Ca^2+^ in the synergid cell reaches a maximum at pollen tube rupture. In *Petunia* and tobacco, the synergid that accepts the pollen tube has high levels of loosely bound Ca^2+^ (Tian and Russel 1997) and membrane-bound Ca^2+^ (Huang and Russel 1992; Tirlapur et al. 1993), which represent exchangeable Ca^2+^ associated with Ca^2+^-binding proteins such as calmodulin or CRT. We postulate that the micropylar-chalazal gradient of *PhCRT* mRNA within the receptive synergid reflects a role of CRT in modulating the local concentration of Ca^2+^ to prevent extra pollen tubes from entering the embryo sac.

Alternatively, an accumulation of *PhCRT* mRNA in the cytoplasm of the receptive synergid after release of the male gametes may reflect a role for Ca^2+^ regulation in positioning of the male gametes for fusion with the female gametes. Beautiful experiments in which fluorescent phalloidin was injected into the embryo sac of *Torenia* clearly showed dramatic changes in the actin organization from anthesis to post-fertilization (Fu et al. 2000). Actin structures called coronas form after anthesis in the receptive synergid and at the interface between egg apparatus cells and vanish after fertilization. These coronas appear to be involved in reception of the pollen tube and migration and deposition of non-motile sperm cells to the fusion site with the female gametes. Studies by Ye et al. (2002) revealed that the two sperm cells deposited in the receptive synergid also have actin filaments oriented along their long axes. Thus, it is likely that transport of the sperm cells to the fusion site depends on actin-myosin interactions within the embryo sac, which in turn requires Ca^2+^. Actin dynamics is a Ca^2+^-dependent process, and the activities of many actin-binding proteins are regulated by Ca^2+^. Therefore, maintenance of an optimal Ca^2+^ environment within the embryo sac is essential for fertilization; the Ca^2+^-buffering protein CRT is an excellent candidate to fulfill this role.

*PhCRT* mRNA transcripts were also detected within the two sperm cells. Although enhancement of *CRT* expression was previously revealed in maize sperm cells (Williams et al. 1997), the possible role of CRT in plant male gametes remains unclear, and further experiments are needed to explain this phenomenon.

### *PhCRT* is highly expressed during pistil transmitting tract maturation and pollen tube outgrowth on the stigma

This paper reports the first link between elevated *CRT* expression and pistil transmitting tract maturation. Both our Northern blots and FISH revealed that the highest level of *PhCRT* expression was in the unpollinated pistil 1 day before anthesis, and that the levels of mRNA were higher in stigma–style fragments of UPI pistils than in the same parts of UPM pistils. In addition, we observed higher abundance of *PhCRT* transcripts in the cytoplasm and nuclei of sitt secretory cells before anthesis. Together, these results suggest that *PhCRT* mRNA transcription starts before the exudate appears on the stigma surface. Similarly, CRT is highly abundant in other plant tissues that are secretory in nature, such as the secreting nectaries in *Arabidopsis* flowers (Nelson et al. 1997), the inner layer of the fertilized ovule (called endothelium) in *Nicotiana* (Borisjuk et al. 1998), and the active tapetum in *Nicotiana* anthers (Nardi et al. 2006). Both the tapetum and the endothelium are involved in synthesis of nutrients for the developing pollen or embryo, whereas the pistil transmitting tract cells produce nutrients for growing pollen tubes. Therefore, we postulate that the secretory sitt (and probably also sytt) cells in *Petunia*, like the endothelium cells and the tapetum cells, require an active biosynthetic and secretory apparatus and rely on CRT as a molecular chaperone to achieve high secretory activity.

We observed a high level of *PhCRT* expression in the upper part of the pistil at the PP1 stage, when pollen germinate and tubes outgrow on the receptive stigma. FISH labeling indicated that the mRNA was mainly localized uniformly in the cytoplasm of the hydrated and germinated pollen as well as in growing pollen tubes. Previously, we showed a similar distribution of *CRT* mRNA in *Haemanthus* germinating pollen and growing tubes in vitro (Lenartowska et al. 2009). The signal was weaker and less specific than in *Petunia* however, most likely owing to the fact that we did not have a *Haemanthus*-specific probe. One explanation for the high level of *CRT* mRNA in germinated pollen and growing tubes might be that the chaperone activity of CRT facilitates the high rate of protein synthesis required for this fast tip-growing cell. Alternatively, CRT’s Ca^2+^-buffering properties may contribute to the maintenance of cytoplasmic Ca^2+^ homeostasis, which is critical for pollen tube growth (see review by Ge et al. 2007).

An important question is whether transcription is required in a pollen tube while it is growing. It is commonly accepted that most plants accumulate mRNAs and proteins in their mature pollen until the time of germination and thus do not require transcription during elongation. For example, recent data show that most of the RNAs required for germination and pollen tube growth are stored in the mature pollen grain in *Petunia*, so de novo transcription may not be necessary (Ishimizu et al. 2010). However, in vitro studies have suggested that de novo mRNA synthesis does occur during pollen germination and tube elongation (see review by Huang et al. 2011). We observed *PhCRT* mRNA in the vegetative nucleus of germinating pollen and very short pollen tubes, but not *PhCRT* mRNA in the nuclei of hydrated pollen and longer tubes. This result is consistent with our previous in vitro studies in *Haemanthus* (Lenartowska et al. 2009) and suggests that transcription is active during pollen germination and the start of tube outgrowth, but then is silent during pollen tube elongation.

Taken together, we conclude that *PhCRT* is expressed during multiple steps of plant reproduction: pistil transmitting tract maturation, pollen germination and tube outgrowth, fertilization, and early embryogenesis. We speculate that CRT’s molecular chaperone and Ca^2+^-buffering activities facilitate these processes, which require high rates of protein synthesis and careful regulation of Ca^2+^ homeostasis.

## Abbreviations

Ca^2+^: Calcium ions
*CRT*: Calreticulin gene
CRT: Calreticulin protein
ER: Endoplasmic reticulum
FISH: Fluorescent in situ hybridization
M-MLV: Moloney murine leukemia virus
MOPS: 3-(*N*-morpholino)propanesulfonic acid
PP1: First stage of the progamic phase
PP2: Second stage of the progamic phase
PP3: Third stage of the progamic stage
Sitt: Stigma transmitting tract
Sytt: Style transmitting tract
UPI: Unpollinated immature pistil
UPM: Unpollinated mature pistil
